# Adverse Events of Antiepileptic Drugs Using Indonesian Version of Liverpool Adverse Events Profile

**DOI:** 10.1155/2018/8490639

**Published:** 2018-11-25

**Authors:** Astri Budikayanti, Lubna Muhammad Qadri, Zakiah Syeban, Luh Ari Indrawati, Fitri Octaviana

**Affiliations:** Department of Neurology, Faculty of Medicine, Universitas Indonesia/Cipto Mangunkusumo National Referral General Hospital, Jakarta, Indonesia

## Abstract

**Introduction:**

Adverse events (AEs) associated with antiepileptic drugs (AEDs) affect people with epilepsy's (PWE) quality of life. A study conducted in 15 European countries showed that the AEs prevalence of AEDs in PWE was up to 80%. To date, there are no validated screening instruments to detect AEs of AEDs in Indonesian PWE. Therefore its epidemiology is currently unknown. This study aimed to validate the Indonesian version of Liverpool Adverse Events Profile (LAEP), consequently increasing physicians' awareness toward the probability of AEs and its necessary evaluation. Furthermore, this study was intended to determine the AEs prevalence of AEDs in Indonesian PWE.

**Methods:**

The questionnaire was translated from English into Indonesian version. The validity and reliability were tested using Spearman correlation and Cronbach's alpha measurement. An observational cross-sectional study was carried out on consecutive PWE in outpatient clinic, Cipto Mangunkusumo Hospital. We analyzed duration of epilepsy, onset of epilepsy, seizure frequency, type of epilepsy, etiology and epilepsy syndrome, number of AEDs, duration of AED use, and comorbidity.

**Results:**

All of the 19 items in the questionnaire were valid, with correlation coefficient ranging from 0.465 to 0.690 (moderate-strong correlation). Cronbach's alpha value was 0.846 (good consistency). The total of 90 subjects were enrolled with 91% screened as having AEs using LAEP questionnaire. The most common AEs were tiredness (67.8%), sleepiness (66.7%), memory problems (62.2%), and difficulty in concentrating (56.7%). The only clinical variable that influenced AEs was polytherapy.

**Conclusion:**

The Indonesian version of LAEP was a valid and reliable instrument to screen AE of AEDs in PWE. Almost all the subjects in this study were suspected having AEs. Polytherapy was the independent factor of AE.

## 1. Introduction

Epilepsy is a disorder of the brain characterized by an enduring predisposition to generate epileptic seizures and by the neurobiologic, cognitive, psychological, and social consequences of this condition [1]. Antiepileptic drugs (AEDs) treatment is the mainstay of management of epilepsies. The ultimate goal of AEDs therapy is to restore a normal health-related quality of life, which primarily depends on the achievement of sustained seizure freedom without clinically disabling adverse events (AEs) [2, 3].

Data from cross-sectional studies shows up to 80% of people with epilepsy taking AEDs experience AEs. [4] AEs are the leading cause of treatment failure with AEDs. AEs result in negative effects in patient adherence as well as early treatment discontinuation in up to 25% of patients. Moreover, AEs are the main source of disability, morbidity, and mortality [5–7]. Studies have shown that quality of life was related to reported AEs and tolerability of AEDs [4, 8]. In epilepsy care, the detection and minimization of AEs related to treatment play an important role. An active approach using screening measures such as self-reported questionnaires allows detection of larger number of patients with AEs [7, 9]. Spontaneous report identifies AEs in only 10-40% of individuals with epilepsy, whereas the use of structured screening method identifies 60-90% or higher [5, 6]. Gilliam et al. in their study reported that systematic screening with a self-reported instrument allows reduction of AEs, and the management of the reported AEs was related to improvement of patients' quality of life [9].

The Liverpool Adverse Events Profile (LAEP) was developed in 1994 by Baker et al. to assess patients' perception of the AEs of their AEDs. LAEP is a self-administered, epilepsy-specific, 19-item questionnaire. It is an internationally used instrument, already translated and validated in Spain, Taiwan, and Brazil [8–11].

To date, there is no validated instrument in measuring the presence and impact of AEs for Indonesian PWE. This study aimed to adapt and validate the Indonesian version of LAEP as a screening tool to detect the presence of AEs of AEDs in PWE. Hopefully, this screening tool can increase physicians' awareness and further evaluation of the administered AEDs. Moreover, this study was also expected to describe the prevalence of AEs of AEDs in PWE and its related factors.

## 2. Methods

### 2.1. Translation of the Liverpool Adverse Events Profile

The process of translation and adaptation into Indonesian language was initiated after permission was obtained from the Liverpool Group (G.Baker) as the developer of LAEP questionnaire. The translation and adaptation steps were conducted using the steps described by the World Health Organization (WHO), which include the following phases: (1) translating the original version of LAEP into Bahasa Indonesia by two independent, qualified bilingual translator; (2) evaluation of the translated questionnaire by expert panels consisting of 4 epileptologists in Cipto Mangunkusumo Hospital; (3) pretesting of the questionnaire to a minimum of 10 PWE; (4) evaluation of the pretesting results and improvement by the panel; (5) backtranslation into English by two other independent, bilingual qualified translators; (6) establishment of the final version and validation study [12, 13].

### 2.2. Validity and Reliability Study

The final version was given to a group of consecutive PWE in outpatient clinic, Cipto Mangunkusumo Hospital. Patients older than 18 years with a confirmed diagnosis of epilepsy for at least 3 months who were taking AEDs at a stable dose for at least 1 month and were able to understand and answer the question items by themselves were included in the study. Patients with mental retardation or having psychotic disorders were excluded.

Validity and reliability were analyzed statistically using SPSS 20.0. Validity was assessed by estimating the correlation between the LAEP items and overall scores in a group of patients using Spearman correlation test. The internal consistency reliability was assessed by estimating Cronbach's alpha coefficient. Values >0.700 are conventionally considered acceptable.

### 2.3. Prevalence of Adverse Events Analysis

PWE fulfilling research criteria in outpatient clinic, Cipto Mangunkusumo Hospital, during 2016 were collected consecutively. Subjects independently completed the Indonesian version of the LAEP. Each item was assessed on a 4-point Likert scale and a global summary score was calculated, ranging from 19 (low prevalence and severity of AEs) to 76 (high prevalence and severity of AEs) [8–10]. Demographic data (age, gender, education level, and marital status) and clinical data (duration of epilepsy onset of epilepsy, frequency of seizure, type of epilepsy, etiology and epilepsy syndrome, number of AEDs, AEDs duration, and comorbidity) were also collected.

Statistical analysis was performed using the SPSS 20.0. Data were subsequently arranged in a frequency distribution table or cross-table in accordance with the study purpose.* p *values less than 0.05 were considered statistically significant. Statistical analysis was performed using chi-square test, Fisher's exact test, and Kolmogorov-Smirnov test as an alternative.

### 2.4. Compliance with Ethics Guideline

Subjects in this study were recruited after gaining approval from the ethics committee and written informed consents for the study were obtained. The ethical approval was granted from Ethical Committee, Faculty of Medicine, Universitas Indonesia.

## 3. Results

### 3.1. Cultural Adaptation

The final Indonesian version of LAEP was translated and culturally adapted using WHO steps. Some items needed additional information and were linguistically adapted to clarify their meaning. The item “feeling of aggression” was changed to “easily getting angry.” The item “depression” required modification and became “depression (feeling guilty, sad, difficulty in finding happiness).” Details are provided in the supplementary material.

### 3.2. Validity and Reliability Analysis

The total of 30 subjects were recruited for validation and reliability study as to maximize the result's significance. Subjects characteristics are depicted in Table 1. Spearman correlation was used to analyze the correlation between the LAEP variables and overall scores. All of the 19 items of the questionnaire were valid, with correlation coefficient ranging from 0.465 to 0.690 (moderate to strong correlation) and significance level less than 0.01. Internal consistency of the overall score as measured with Cronbach's alpha coefficient was 0.846.

### 3.3. Demographic and Clinical Characteristic

For prevalence and related factors analysis, the total of 90 subjects were enrolled. The median value of age was 32.5 years old (range 18-60 years old). Majority of the subjects were female (55.6%) and had an educational level of high school graduate (42.2%). The median value of epilepsy onset was 17 years old (range 2-58 years old) and duration of epilepsy was 8.5 years (range 6 month to 46 years).

Most subjects had been seizure-free in one month (72.2%), had focal seizure type (96.7%), had epilepsy (68.9%), had temporal epilepsy syndrome (57.8%), and were using monotherapy in fifty-six subjects (62.2%). Subjects' demographic and clinical characteristic were summarized in Table 2.

Carbamazepine was the most common AED used (32.2%), followed by levetiracetam (25.6%), valproic acid (24.4%), phenytoin (22.2%), and clobazam (20%).

### 3.4. Prevalence of Adverse Events, the Indonesian Version of LAEP

In this study, the total of 91% subjects were screened as having AEs. The median value of LAEP score was 31.5 (range 19-49).

**Figure 1:**
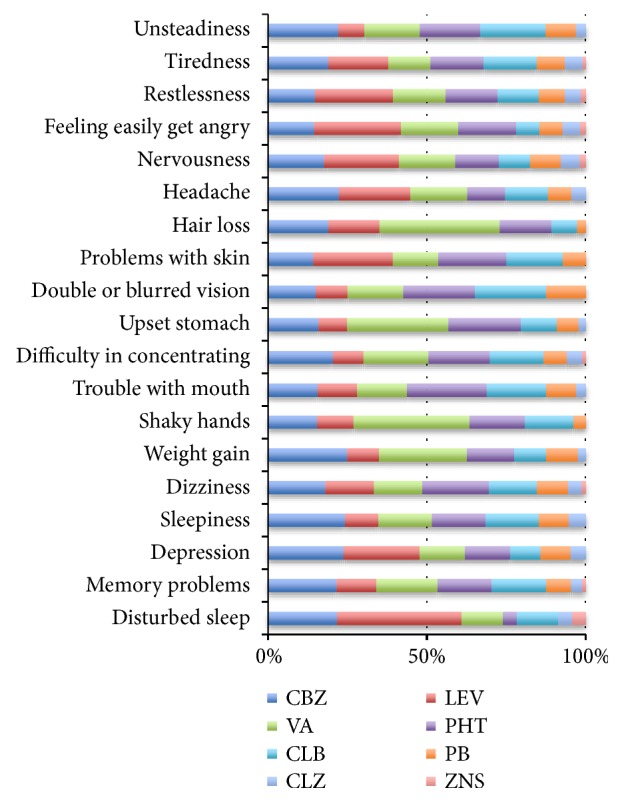
Type of AED treatment and the occurrence of AE.

Mostly reported AEs were tiredness (67.8%), sleepiness (66.7%), memory problems (62.2%), and difficulty in concentrating (56.7%). Subjects taking carbamazepine complained of sleepiness (38.3%), unsteadiness (37.8%), weight gain (35.7%), difficulty in concentrating (33.3%), memory problems (33.9%), and headache (33.3%). Patients taking levetiracetam experienced feeling easily getting angry (38.5%), restlessness (36.3%), being depressed (33.3%), nervousness and/or agitation (35.3%), skin problems (35%), and disturbed sleep (52.9%). Subjects taking valproic acid complained of shaky hands (57.6%), hair loss (56%), upset stomach (48.3%), and weight gain (39.3%).  Figure 1 summarized the type of AEDs treatment and the occurrence of AEs.

We also tried to determine dose-related AEs for each AEDs. Subjects taking carbamazepine complained of sleepiness at mean dose of 686.96 ± 338.18 mg, unsteadiness at 757.14 ± 376.14 mg, weight gain at 760 ± 474.81 mg, difficulty in concentrating at 741.18 ± 316.34 mg, memory problems at 692.11 ± 360.66 mg, and headache at mean dose of 713.33 ± 372.95 mg. Subjects taking levetiracetam experienced feeling easily getting angry, restless, and depressed at the dose of 1.000 mg, while at the minimum dose of 250 mg they already felt nervousness and/or agitation, having problems with skin, and disturbed sleep. Subjects with valproic acid had shaky hands at mean dose 894.74 ± 336.61 mg, hair loss at mean dose 875 ± 235.13 mg, upset stomach at mean dose 875 ± 254.76 mg, and weight gain at mean dose 1,000 ± 273.86 mg. Subjects taking phenytoin at the minimum dose of 200 mg had dizziness and oral or gum problems while at 225 mg subjects had double or blur vision.

Subjects undergoing polytherapy tended to have AEs more than those undergoing monotherapy while epilepsy duration, seizure frequency, epilepsy type, epilepsy etiology and syndrome, numbers of AEDs, AEDs duration, type of AEDs, and comorbidity did not show any relation with AEs (Table 3).

## 4. Discussion

The Indonesian version of LAEP was translated and culturally adapted according to the WHO standard guideline [13]. There were only 2 items requiring linguistic adjustment to make the questionnaire more acceptable for Indonesian PWE. The final version of Indonesian LAEP was obtained and considered to be as valid and reliable as the original version, since the translation and adaptation steps were also evaluated and retained the validity of the questionnaire items [12]. Statistical assessment was also conducted to see the consistency and reliability of the questionnaire. All of the 19 items were valid and reliable with the correlation coefficient of each item falling between moderate and strong category (p<0.01). Internal consistency of the overall score as measured with Cronbach's alpha coefficient was 0.846. Validation of LAEP in Spain by Carreno et al. had the same internal consistency (Cronbach's alpha 0.84) [14]. Meanwhile Brazil, Korea, and Bulgaria's Cronbach's alpha value were 0.90, 0.90, and 0.86 respectively [15–18]. Nevertheless, Cronbach's alpha values in these countries were still equal.

The prevalence of AEs in this study was 91%. There are several factors that have been known to contribute to the high prevalence of AEs, such as the use of structured screening instrument, polytherapy, and the administration of older AED [19]. LAEP as a self-reported screening tool was suggested to screen higher rates of adverse events compared to spontaneous reports [8, 9]. In a cross-sectional study undertaken in 56 epilepsy outpatient clinics in Spain, adverse events of antiepileptic drugs were reported in 34% of patients when assessed by spontaneous reporting and 65% when a checklist was used [5, 9]. In an Italian multicentre study of 809 consecutive patients with drug-resistant epilepsy, the prevalence of adverse events identified by a validated screening method was almost three times greater than that detected with an unstructured interview (96% vs 37%) [3, 5]. In this study, cognitive complaints, emotional complaints, weight changes, and hair loss were highly reported. These symptoms were likely to be neglected by patients and were not considered a particular problem as an AE. Moreover, regular doctor visits may be too short in time and thus these complaints were seldomly described [8]. Therefore, the use of self-report structured screening instrument yields higher prevalence of AEs.

Previous study also reported polytherapy increased the risk of AEs. This study showed that 37.8% of subjects undergoing polytherapy and subjects with polytherapy had 1.167 times higher risk of AEs than subjects on monotherapy. This result was consistent with the validation study in England, Taiwan, and Brazil [15, 16]. Andrew et al. in his study also concluded that subjects on polytherapy had significantly higher LAEP scores than subjects on monotherapy [20]. It is postulated that the administration of polytherapy increases patients' risk of developing AEs due to the likelihood of drug interactions. It is clear that drug interaction might become one of the most important considerations in AEDs administration as the pharmacokinetic of one drug could alter the absorption, metabolism, protein binding process, or the excretion of the other drug. This will change the efficacy of each AED and increase the risk of AEs. Older generation AEDs like carbamazepine, phenytoin, phenobarbital, and valproic acid are metabolized by the cytochrome P450 in the liver and interfere with the enzyme activities. Therefore, patients taking older generation AEDs have a higher risk of developing AEs. Lastly, the administration of drugs with overlapping mechanism will tend to cause pharmacodynamics interactions and resulting in higher risk for AEs. In this study, 91.2% subjects on polytherapy were using carbamazepine, phenytoin and valproic acid with 45.2% of them using two of these AEDs.

Other clinical characteristics were also statistically analyzed, yet they did not show any significant correlation with the number of AEs. Therefore, the high prevalence of AEs in this study resulted from the use of polytherapy and LAEP as a self-report screening instrument.

The most reported AEs in this study were CNS related symptoms which consisted of restlessness, sleepiness, memory problems, and difficulty in concentrating. These could be associated with the most frequently used AEDs, carbamazepine, levetiracetam, valproic acid, and phenytoin. Similar results were reported in study by Baker et al., Chen et al., and Martins et al. [4, 15, 16]. Hirsch et al. reported the most common AEs in levetiracetam were behavioral changes and psychiatric AEs [21]. Results from Hirsch et al. were consistent with this study. In this study, the most AEs in the use of levetiracetam were restlessness, feeling easily getting angry, nervousness and or agitation, and depression. The main AEDs mechanism of action is averting the neuronal excitability underlying the occurrence of seizure; therefore the most reported AEs are related to CNS. The AEs in subjects on carbamazepine occurred below the recommended maintenance dose. This might be caused by narrow therapeutic index of carbamazepine, resulting in the presence of AE with just a small dose of carbamazepine

All older generation antiepileptic drugs, particularly carbamazepine, phenytoin, and benzodiazepines, are associated with substantial risk of coordination disturbances. Coordination disturbances include dizziness, unsteadiness, vertigo, imbalance, ataxia, nystagmus, diplopia, and tremor [5]. In this study the most AEs caused by phenytoin were double or blurred vision and dizziness.

Seizure frequency was not related to AEs in this study. This finding was in accordance with other studies that found no relationship between seizure frequency and the LAEP or AEs [3, 8]. A study by Martin et al. however showed a correlation between seizure frequency and LAEP scores. In our opinion, this data was due to the fact that half of the subjects in the study were categorized as difficult-to-treat epilepsies, such as in mesial temporal sclerosis and long duration juvenile myoclonic epilepsy [16].

The Indonesian version of LAEP was confirmed to be a reliable and valid instrument in assessing AEs of AEDs in PWE. Most subjects in this study (91%) were screened as having AEs, yet a thorough examination needs to be performed in order to confirm this finding. Polytherapy was the related factor of AEs of AEDs.

## Figures and Tables

**Table 1:** Subjects Demographic Features for Validation Study (n=30).

**Variable**	**n (**%**)**
Sex	
(i) Woman	18 (60)
(ii) Men	12 (40)
Educational background	
(i) Primary	9 (30)
(ii) High school	16 (53.3)
(iii) University	5 (16.7)
Occupation	
(i) Working	12 (40)
(ii) Not working	18 (60)
Marital status	
(i) Married	14 (46.7)
(ii) Single	16 (53.3)

**Table 2:** Subjects Demographic and Clinical Characteristic (n=90).

**Variable**	**n (**%**)**
**Gender**	
Female	50 (55.6)
Male	40 (44.4)
**Education level**	
Junior high school	32 (35.6)
Senior high school	38 (42.2)
Academy	20 (22.2)
**Seizure frequency**	
Seizure free	65 (72.2)
Uncontrolled seizure	25 (27.8)
**Seizure type**	
Focal seizure	87 (96.7)
Generalized seizure	3 (3.3)
**Epilepsy etiology**	
Structural	62 (68.9)
Unknown Etiology	25 (27.8)
Genetic	3 (3.3)
**Epilepsy syndrome**	
Extratemporal	35 (38.9)
Temporal	52 (57.8)
Generalized	3 (3.3)
**Number of AED**	
Polytherapy	34 (37.8)
Monotherapy	56 (62.2)
**Comorbid**	
Yes	21 (23.2)
No	69 (76.7)

**Table 3:** Clinical characteristic and their relation to AEs (n=90).

**Variables**	**Adverse event**				
	**Yes**	**No **	***p***	**OR**	**95% confidence interval**
	**n (%)**	**n (%)**			**Lower-Upper**
**Gender**					
(i) **Female**	47 (94)	3 (6)	0.458^*∗*^	2.238	0.501 – 9.998
(ii) **Male**	35 (87.5)	5 (12.5)			
**Education**					
(i) Junior High School	29 (90.6)	3 (9.4)	1.000^*∗∗*^	0.829	0.155 – 4.420
(ii) Senior High School	35 (92.1)	3 (7.9)			
(iii) Academy	18 (90)	2 (10)		1.296	0.198 – 8.473
**Employment**					
(i) Employed	27 (87.1)	4 (12.9)	0.439^*∗*^	0.491	0.114 – 2.115
(ii) Unemployed	55 (93.2)	4 (6.8)			
**Marital Status**					
(i) Married	39 (88.6)	5 (11.4)	0.480^*∗*^	0.544	0.122 – 2.428
(ii) Unmarried	43 (93.5)	3 (6.5)			
**Epilepsy duration**					
(i) ≥ 8.5 years	42 (93.3)	3 (6.7)	0.714^*∗*^	1.750	0.392 – 7.807
(ii) < 8.5 years	40 (88.9)	5 (11.1)			
**Seizure frequency**					
(i) Uncontrolled seizure	25 (100)	0 (0)	0.100^*∗*^	1.140	1.041 – 1.249
(ii) Seizure free	57 (87.7)	8 (12.3)			
**Seizure type**					
(i) Focal seizure	80 (92)	7 (8)	0.246^*∗*^	5.714	0.459 – 71.142
(ii) Generalized seizure	2 (66.7)	1 (33.3)			
**Epilepsy etiology**					
(i) Structural	57 (91.9)	5 (8.1)	1.000^*∗∗*^	0.991	0.179 – 5.480
(ii) Unknown Etiology	23 (92)	2 (8)			
(iii) Genetic	2 (66.7)	1 (33.3)		5.750	0.349 – 94.725
**Epilepsy syndrome**					
(i) Extratemporal	33 (94.3)	2 (5.7)	0.996^*∗∗*^	1.755	0.321 – 9.601
(ii) Temporal	47 (90.4)	5 (9.6)			
(iii) Generalized	2 (66.7)	1 (33.3)		4.700	0.359 – 61.497
**Number of AED**					
(i) Polytherapy	34 (100)	0 (0)	**0.022** ^*∗*^	1.167	1.048 – 1.298
(ii) Monotherapy	48 (85.7)	8 (14.3)			
**AED duration**					
(i) ≥ 1 year	66 (91.7)	6 (8.3)	0.658^*∗*^	1.375	0.253 – 7.459
(ii) < 1 year	16 (88.9)	2 (11.1)			
**Type of AED**					
(i) Carbamazepine	25 (86.2)	4 (13.8)	0.266^*∗*^	0.439	0.102 – 1.895
(ii) Levetiracetam	22 (95.7)	1 (4.3)	0.674^*∗*^	2.567	0.299 – 22.067
(iii) Valproic acid	21 (95.5)	1 (4.5)	0.674^*∗*^	2.410	0.280 – 20.754
(iv) Phenytoin	20 (100)	0 (0)	0,191^*∗*^	1.129	1.038 – 1.228
(v) Clobazam	17 (94.4)	1 (5.6)	1.000^*∗*^	1.831	0.211 – 15.911
(vi) Phenobarbital	9 (100)	0 (0)	1.000^*∗*^	1.110	1.032 – 1.193
(vii) Clonazepam	5 (100)	0 (0)	1,000^*∗*^	1.104	1.031 – 1.182
(viii) Zonisamid	1 (50)	1 (50)	0.171^*∗*^	0.086	0.005 – 1.535
**Comorbid**					
(i) Yes	20 (95.2)	1 (4.8)	0.675^*∗*^	2.258	0.262-19.483
(ii) No	62 (89.9)	7 (10.1)			

^*∗*^Fisher's Exact test. ^*∗∗*^Kolmogorov-Smirnov test.

## Data Availability

The datasets generated during and/or analyzed during the current study are available from the corresponding author on reasonable request.
